# RNAi Efficiency through dsRNA Injection Is Enhanced by Knockdown of dsRNA Nucleases in the Fall Webworm, *Hyphantria cunea* (Lepidoptera: Arctiidae)

**DOI:** 10.3390/ijms23116182

**Published:** 2022-05-31

**Authors:** Xun Zhang, Zhizhi Fan, Qinghua Wang, Xiangbo Kong, Fu Liu, Jiaxing Fang, Sufang Zhang, Zhen Zhang

**Affiliations:** Key Laboratory of Forest Protection of National Forestry and Grassland Administration, Ecology and Nature Conservation Institute, Chinese Academy of Forestry, Beijing 100091, China; zhx3355@126.com (X.Z.); 18234494745@163.com (Z.F.); wqh633@caf.ac.cn (Q.W.); xbkong@sina.com (X.K.); liufu2006@163.com (F.L.); fjxinsect@163.com (J.F.)

**Keywords:** dsRNase, dsRNA degradation, RNA interference, RNAi efficiency, *Hyphantria cunea*

## Abstract

RNA interference (RNAi) technology is a promising approach used in pest control. The efficiency of RNAi varies considerably among different insect species, and growing evidence suggests that degradation of double-stranded RNA (dsRNA) prior to uptake is an important factor that limits RNAi efficiency in insects. Our recent work on fall webworm (*Hyphantria cunea*), an important invasive pest in China, showed a relatively low silencing efficiency of RNAi through dsRNA injection, which is considered the most feasible dsRNA delivery method for inducing RNAi, and the factors involved in the mechanism remain unknown. Herein, we first detected the dsRNA-degrading activity in the hemolymph and gut content of *H. cunea* in ex vivo assays and observed rapid degradation of dsRNA, especially in the hemolymph, which was complete within only 10 min. To determine whether dsRNA degradation could contribute to the low effectiveness of RNAi in *H. cunea*, four dsRNA nuclease (*dsRNase*) genes, *HcdsRNase1, HcdsRNase2, HcdsRNase3*, and *HcdsRNase4,* were identified by homology searching against the *H. cunea* transcriptome database, and their transcript levels were subsequently investigated in different tissues, developmental stages, and after dsRNA injection. Our results show that *HcdsRNases* are highly expressed mainly in gut tissues and hemolymph, and the expression of *HcdsRNase3* and *HcdsRNase4* were significantly upregulated by dsGFP induction. RNAi-of-RNAi studies, using *HcCht5* as a reporter gene, demonstrated that silencing *HcdsRNase3* and *HcdsRNase4* significantly increases RNAi efficacy via dsHcCht5 injection, and co-silencing these two *HcdsRNase* genes results in a more significant improvement in efficacy. These results confirm that the RNAi efficacy in *H. cunea* through dsRNA injection is certainly impaired by dsRNase activity, and that blocking *HcdsRNases* could potentially improve RNAi, providing a reference for related studies on insects where RNAi has low efficiency.

## 1. Introduction

The fall webworm (*Hyphantria cunea*), a lepidopteran insect belonging to the family Arctiidae, is a worldwide forest pest that originated in North America [1]. It has caused significant economic and ecological damage since first being reported in China in 1979 [2,3]. Ecologically, due to its high fecundity and enhanced survivability, this pest is extremely competitive and a threat to biodiversity [4]. Although various control strategies such as natural predation, microbial intervention, and insecticides have been developed to alleviate the damage caused by *H. cunea* [5,6], effective control of this pest has been difficult. Therefore, development of an efficient and environmentally friendly approach to controlling *H. cunea* is urgently required.

Gene silencing mediated by double-stranded RNA (dsRNA) post-transcriptionally suppresses gene expression in a sequence-specific manner [7,8]. RNAi technology is becoming one of the most promising tools for gene function studies as well as a means of pest management in the field [9,10,11]. However, the efficiency of gene silencing by RNAi in insects varies according to the target taxa, and lepidopteran species have been shown to be particularly recalcitrant to RNAi [12,13,14]. In general, dsRNAs are more likely to elicit an RNAi response when delivered by injection rather than through feeding [15,16]. However, it is difficult to achieve an effective RNAi response by dsRNA injection in *H. cunea* compared with other lepidopterans: for example, 1 μg dsRNA of IIS (insulin/insulin-like growth factor signaling) genes was needed in *Maruca vitrata*, 3 μg dsRNA of *TPS* (trehalose-6-phosphate synthase) gene was needed in *Heortia vitessoides*, and 5 μg dsRNA of *CHS* (chitin synthase) gene was needed in *S. litura*, whereas *H. cunea* required 10 μg dsRNA injection for effective silencing of chitin deacetylase (*CDA*) genes [17,18,19,20]. Our previous work also showed that RNAi via dsRNA injection has a rather modest effect on *H. cunea* larvae. The target chitinase 5 (*HcCht5*) gene maintained about 50% expression after injection with different concentrations of dsHcCht5 [21]. We also failed to induce RNAi in larvae by feeding the gene-specific dsRNA (unpublished data). This low RNAi response in *H. cunea* seriously hinders the application of RNAi-based pest control.

Many factors or molecular mechanisms influencing RNAi efficiency have been proposed, such as endosomal entrapment, malfunction of the core machinery, restricted systemic spread, and the presence of dsRNA nuclease (dsRNase) in body fluids of insects [10,12,22]. Recently, dsRNases are increasingly seen as the major factor responsible for the limited RNAi efficiency owing to their exclusive abilities in dsRNA degradation [23,24,25]. Insect dsRNase was first identified in the midgut fluid of the domestic silk moth (*Bombyx mori*) and contains a signal peptide and a nonspecific endonuclease (NUC) domain [26]. Ex vivo experiments have shown that dsRNase activity varies according to insect order. In coleopteran insects, *dsRNase* genes are mainly expressed in gut [15,27,28], while in hemipteran, dsRNases are active in saliva but not in gut or hemolymph [29,30]. By comparison, dsRNA degradation occurs much more rapidly in hemolymph or gut of lepidopteran insects than those of other orders, such as in the case of *dsRNase* in *Heliothis virescens* and *Spodoptera frugiperda* [8,24,31,32]. Knockdown of *dsRNase* expression greatly improves RNAi efficacy in several insects, including *Bactrocera tryoni*, *Leptinotarsa decemlineata*, and *Nezara viridula* [33,34,35], demonstrating that *dsRNases* are indeed responsible for the limited RNAi efficacy. Until now, few studies have characterized dsRNases in *H. cunea*. Considering the importance of dsRNases in dsRNA degradation and their effect on varying RNAi efficiency, it is necessary to determine whether dsRNases in *H. cunea* are responsible for their lower sensitivity to RNAi.

In the current study, firstly, dsRNA stability in the hemolymph and gut content of *H. cunea* were detected in ex vivo assays. Then, we identified and characterized four *HcdsRNase* genes and investigated their expression profiles in different tissues, development stages, and after dsRNA injection. “RNAi-of-RNAi” injection assays were then performed to determine the contribution of HcdsRNases to RNAi efficacy. We found that separately silencing two of the *dsRNases*, *HcdsRNase3* and *HcdsRNase4,* could improve RNAi efficiency, and co-silencing achieved an enhanced significant promoting effect, suggesting that these two dsRNases mainly contributed to the RNAi recalcitrance observed in *H. cunea.* These results provide a better understanding of the low sensitivity of fall webworm to RNAi.

## 2. Results

### 2.1. Both Hemolymph and Gut Content of H. cunea Rapidly Degrade dsRNA

**Figure 1:**
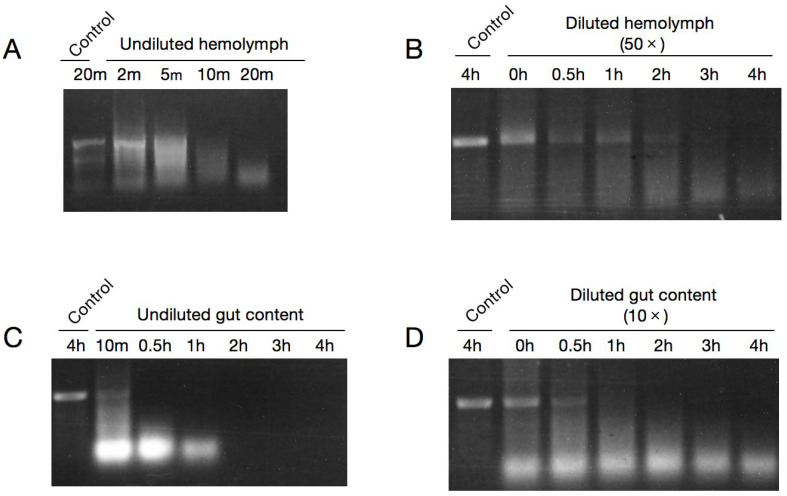
Representative gel images showing that dsRNA was rapidly degraded in either (**A**,**C**) undiluted or (**B**,**D**) diluted hemolymph and gut content collected from 2-day-old fifth-instar larvae. The relative stability of dsGFP was detected by 1% agarose gel electrophoresis after incubation for different time periods in 60 μL tissue extracts (hemolymph or gut content) or nuclease-free water (control). m, minutes; h, hours.

To assess dsRNA stability in hemolymph and gut content, 3 μg dsGFP was incubated with undiluted extracts at 30 °C, and the stability of dsGFP was detected by agarose gel at 10 m, 0.5, 1, 2, 3, and 4 h. We found that the degradation by gut content became visible after 10 min and completed at 2 h (Figure 1C). Surprisingly, degradation by hemolymph was even faster, whereby dsGFP was completely degraded within only 10 min in the undiluted hemolymph (Figure S1). To better evaluate the stability of dsRNA in hemolymph, we set earlier and more intensive time points (2, 5, 10, and 20 min) to examine the degradation of dsGFP in undiluted hemolymph. Results showed that dsGFP was mostly degraded at 5 min and completely degraded at 10 min (Figure 1A). Then, stability of dsGFP in diluted gut content (10×) and diluted hemolymph (50×) were detected respectively. We found that dsGFP was completely degraded by 10-fold diluted gut content within 2 h and by 50-fold diluted hemolymph within 3 h (Figure 1B,D). These results demonstrate the extreme instability of dsRNA in the hemolymph and gut content of *H. cunea*.

### 2.2. Identification and Characterization of Four HcdsRNase Genes from H. cunea

Genome-wide identification of genes encoding *dsRNases* was conducted. By searching the transcriptome data of *H. cunea*, four nucleotide sequences were retrieved encoding the genes *HcdsRNase1, HcdsRNase2, HcdsRNase3,* and *HcdsRNa4*, belonging to the dsRNase subfamily. Among these sequences, the intact open reading frame (ORF) of three genes (*HcdsRNase2*, *HcdsRNase3*, and *HcdsRNase4*) were identified by ORF finder (https://www.ncbi.nlm.nih.gov/orffinder/, accessed on 20 February 2020) and verified by BLASTX search of the NCBI nonredundant protein database. RACE was performed to obtain the full-length cDNA sequences of the *HcdsRNase1*. All *HcdsRNase* genes encoding NUC superfamily members identified from *H. cunea* were confirmed by cloning and sequencing of the complete ORF.

**Figure 2:**
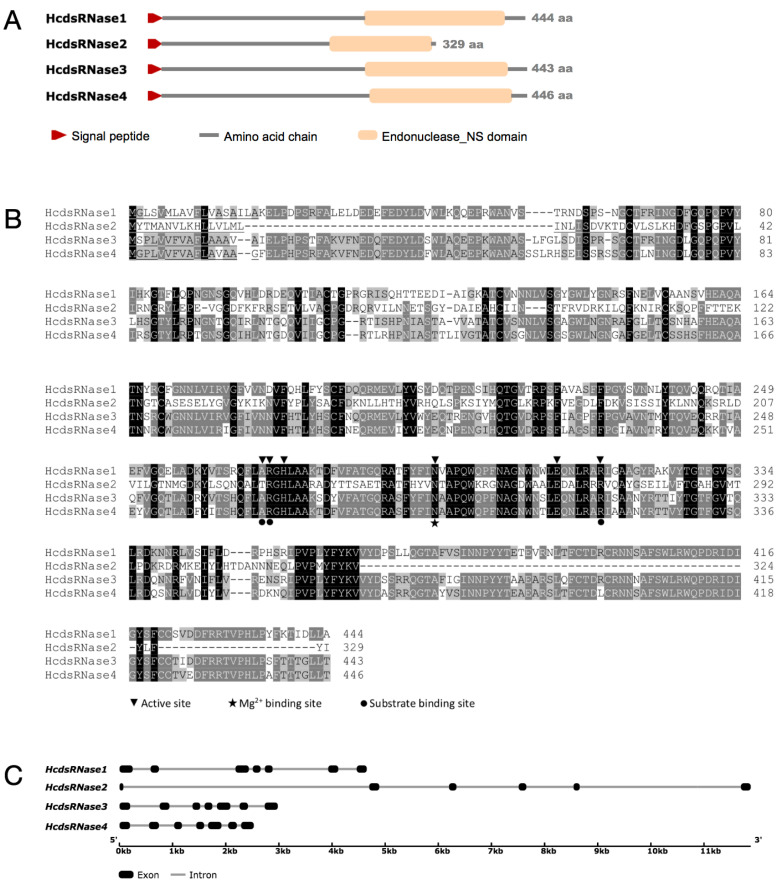
Deduced amino acid sequences analysis and gene structure analysis of HcdsRNases. (**A**) Domain arrangement in the aligned amino acid sequences of HcdsRNases. Red arrows indicate the location of signal peptides, orange boxes represent endonuclease NS domains, and gray lines represent amino acid chains. (**B**) Multiple sequence alignment of deduced HcdsRNase proteins. Residues highlighted in black and gray are conserved and similar, respectively. Signal peptides are underlined. Active sites are marked by triangles, the Mg^2+^ binding site by a star, and substrate binding sites by circles. (**C**) Gene structures of the HcdsRNases. Squares and lines indicate exons and introns, respectively, in individual HcdsRNase genes.

Based on the deduced amino acid sequences, the *HcdsRNases* were found to range within 329–446 amino acids with molecular weights ranging from 37.64 to 50.56 kDa (Table 1). The predicted *pI* values vary from 6.51 to 9.25 (Table 1). All proteins contain a signal peptide comprising 16–25 amino acid residues, which suggests that they are secreted by cells and might have perform some extracellular functions in the body (Table 1, Figure 2A). Sequences of all genes contained the NUC domain, as shown by BLASTP or Pfam matches (Table 1, Figure 2A). Multiple amino acid sequence alignment revealed that the key amino acid residues corresponding to the active site, substrate binding site, and magnesium ion binding site were conserved among *H. cunea* NUC proteins (Figure 2B). By searching the genome database of *H. cunea* (GenBank accession: PKRV00000000.1), we obtained genomic regions of all the four *HcdsRNases*, and the organization of exons and introns was further analyzed. *HcdsRNase1*, *HcdsRNase3*, and *HcdsRNase4* were found to contain seven exons, and *HcdsRNase2* contains six exons (Figure 2C). HcdsRNase3 and HcdsRNase4 show the highest amino acid identity (82.29%) to each other. HcdsRNase1 shows an amino acid identity of 70.49% and 68.30% with HcdsRNase3 and HcdsRNase4, respectively. HcdsRNase2 shows a lower identity of 31.83–34.11% with other HcdsRNases (Table 2).

A phylogenetic tree was constructed using 30 putative insect dsRNase sequences, eight eukaryotic endonuclease G sequences, and three bacterial DNA/RNA non-specific nuclease sequences (Figure S2). The putative insect dsRNase sequence data were retrieved from 18 different insect species that belong to six different major insect orders (Orthoptera, Lepidoptera, Diptera, Coleoptera, Hymenoptera, and Hemiptera), displaying a widespread taxonomic distribution. The HcdsRNase proteins are dsRNase homologs in insects. HcdsRNase1, HcdsRNase3, and HcdsRNase4 clustered in the main Lepidoptera clade, and HcdsRNase2 clustered with the Hemiptera clade (Figure S2).

Amino acid sequences of four *HcdsRNase* genes were deposited in the NCBI database and can be accessed according to the following accession numbers: MZ981769 (HcdsRNase1), MZ981770 (HcdsRNase2), MZ981771 (HcdsRNase3), and MZ981772 (HcdsRNase4).

### 2.3. HcdsRNase Genes Are Expressed in all the Instars of H. cunea Larvae and Are Mainly Functional in Gut and Hemolymph Tissues

**Figure 3:**
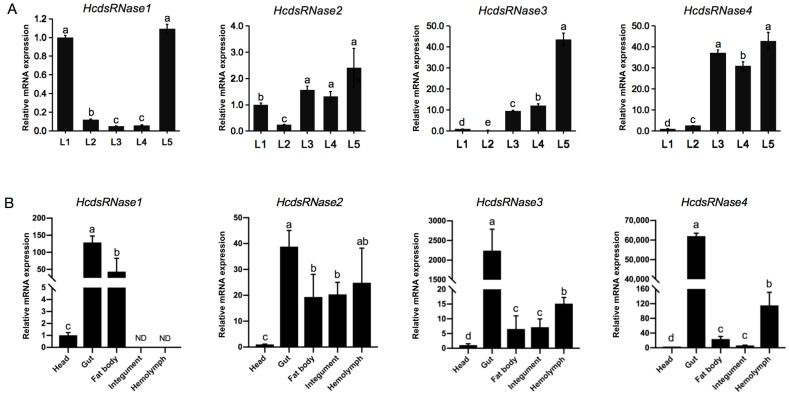
Expression profiles of the four *HcdsRNase* genes in *H. cunea*. (**A**) Relative expression level of *HcdsRNases* at different developmental stages of *H. cunea*. cDNA templates were prepared from total RNA isolated from 3-day-old larvae of each larval stadium (L1–L5). (**B**) Relative expression level of *HcdsRNases* in different tissues or body parts in *H. cunea*. cDNA templates were prepared from total RNA isolated from 2-day-old fifth-instar larvae dissected heads, guts, fat bodies, integuments, and hemolymph. *β-actin* was used as the reference gene. Values are the means ± SE from three replicates. Different letters on columns represent significant differences (*p* < 0.05). ND means nondetectable expression.

To investigate the developmental stage-specific expression of the four *HcdsRNases*, mRNA expression levels in day 3 larvae from the first instar to the fifth instar (L1–L5) were monitored by RT-qPCR. All four *HcdsRNase* genes were detected to be expressed in all instars. *HcdsRNase1* showed high expression in the first and fifth instars, *HcdsRNase2*, *HcdsRNase3*, and *HcdsRNase4* showed low transcript levels in the early stage (L1 and L2) and high levels in the third to fifth instars (L3 to L5) (Figure 3A).

To study the relative transcript levels of *HcdsRNases* in different tissues, total RNAs were isolated from the head, gut, fat body, integument, and midgut tissues of fifth-instar *H. cunea* larvae. Gene transcript levels were then analyzed by RT-qPCR. Results show that expression of the *HcdsRNases* could be detected in all examined tissues except in the case of *HcdsRNase1*, which was not found to be expressed in integument and hemolymph. Note that all the *HcdsRNases* demonstrated low expression in head and significantly high expression in gut, especially *HcdsRNase3* and *HcdsRNase4*. Three of them, *HcdsRNase2, 3,* and *4*, were also highly expressed in hemolymph (Figure 3B). Overall, the *HcdsRNases* show wide expression in various tissues of *H. cunea* and appear mainly functional in the gut and hemolymph.

### 2.4. dsRNA Injection can Induce Expression of HcdsRNase3 and HcdsRNase4

**Figure 4:**
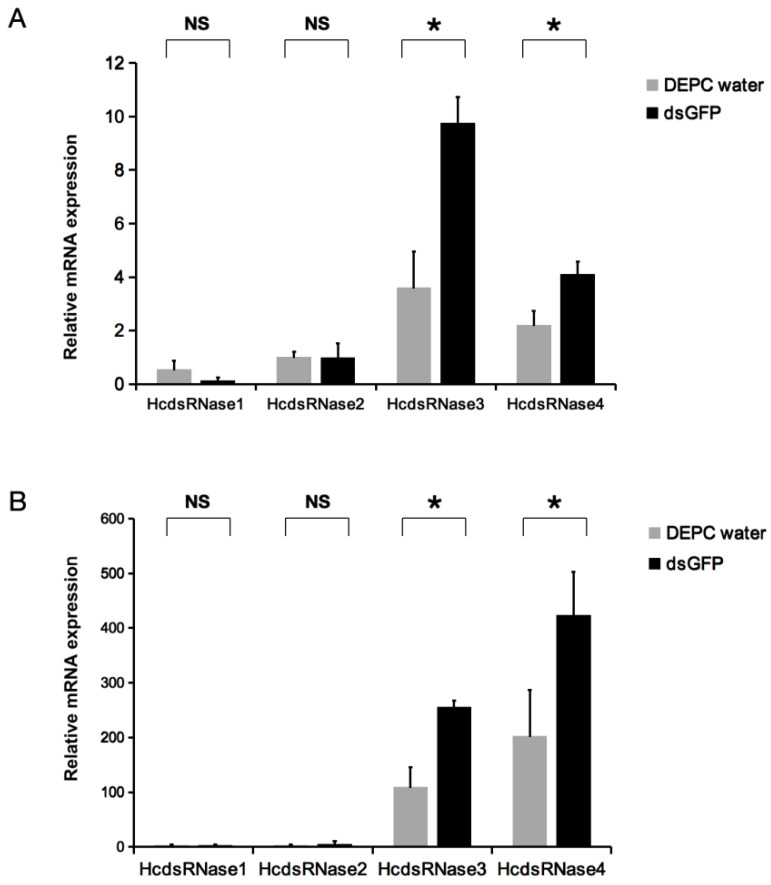
Relative mRNA expression level of the four *HcdsRNase* genes after dsGFP injection at (**A**) 24 h and (**B**) 48 h. Two-day-old fourth-instar larvae were injected with 2 μL of 6 μg dsGFP (black columns, the treatment group) or 2 μL DEPC water (gray columns, the control group). Error bars represent the standard error of the calculated means based on four replicates. * Asterisks indicate significant differences (*p* < 0.05). NS means no significant differences.

In order to understand the effect of dsRNA injection on the expression levels of *HcdsRNases*, we investigated the short-term transcriptional response of *HcdsRNases* after dsGFP injection. Two-day-old fourth-instar larvae were injected with dsGFP, and the expression levels of the four *HcdsRNase* genes were detected by RT-qPCR at 24 and 48 h post treatment. We discovered that there was no significant difference in the expression of *HcdsRNase1* and *HcdsRNase2* at 24 and 48 h. However, *HcdsRNase3* and *HcdsRNase4* were significantly highly expressed in the dsGFP-treated group compared to the control (DEPC water) both at 24 and 48 h (Figure 4A,B). Further tissue expression detection showed that these two *HcdsRNases* were mainly upregulated in the gut tissues (Figure S3). These results indicate that the expression level of *HcdsRNase3* and *HcdsRNase4* can be upregulated by dsRNA injection.

### 2.5. HcdsRNase Genes can Be Effectively Silenced by Injecting the Corresponding dsRNAs

**Figure 5:**
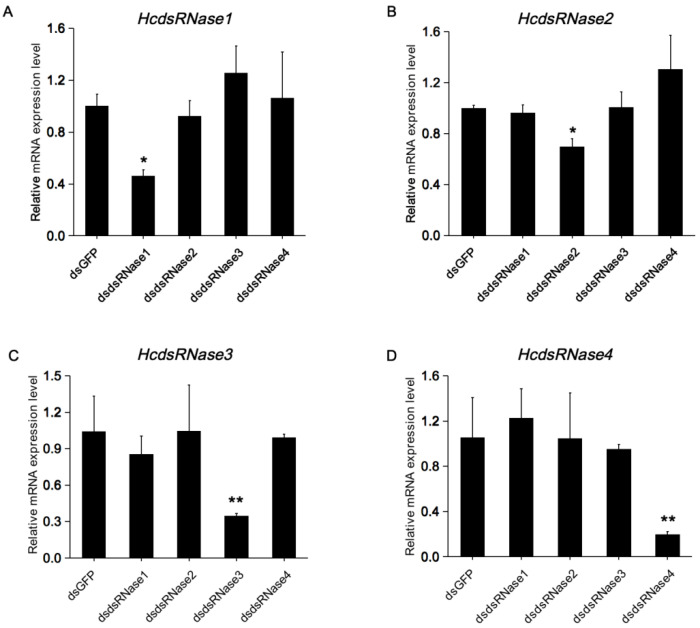
Gene silencing of (**A**) *HcdsRNase1*, (**B**) *HcdsRNase2*, (**C**) *HcdsRNase3*, and (**D**) *HcdsRNase4* after injection of the corresponding dsdsRNase. cDNA templates employed for RT-qPCR were prepared from total RNA extracted from larvae at 48 h after injection with dsRNAs corresponding to the four *HcdsRNases* and *GFP* controls. Error bars represent the standard error of the calculated means based on four replicates. Asterisks indicate significant differences according to Student’s *t*-test (* *p* < 0.05; ** *p* < 0.01).

To evaluate whether silencing of *HcdsRNases* can improve the RNAi efficacy in *H. cunea*, RNAi silencing of the four *HcdsRNase* genes was firstly carried out by individual injection of dsRNase-specific dsRNA (dsdsRNase). The expression level of the *HcdsRNase* genes were detected 48 h post treatment. Results show that the four *HcdsRNases* were successfully silenced by the corresponding dsRNAs, and no off-target effects occurred (Figure 5A–D). Therefore, dsdsRNase injection can effectively downregulate the corresponding *HcdsRNase* gene expression level in *H. cunea*.

### 2.6. HcdsRNase3 and HcdsRNase4 Inhibits RNAi Efficacy

**Figure 6:**
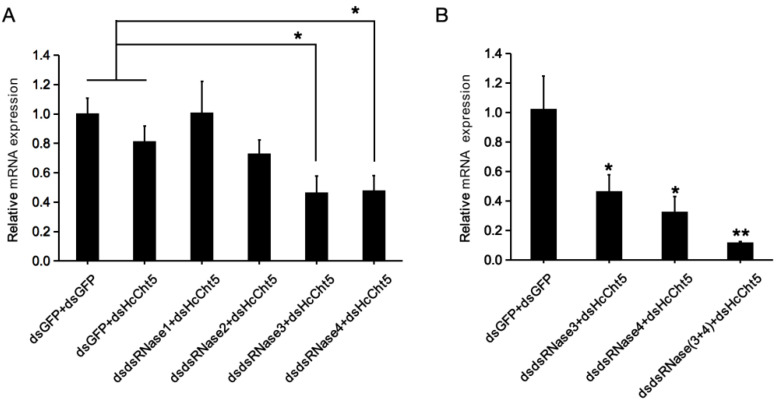
RNAi effects of dsRNase-silenced *H. cunea* larvae by injection of the dsRNA targeting gene *HcCht5*. (**A**) RNAi silencing of *HcCht5* after individual silencing of *HcdsRNase1, HcdsRNase2, HcdsRNase3,* and *HcdsRNase4*. The 2-day-old fourth-instar larvae were injected with 6 μg of *GFP-, dsRNase1-, dsRNase2-, dsRNase3-,* or *dsRNase4-*dsRNAs for 48 h, and then injected with 2 μg dsGFP or dsHcCht5. Transcription levels of *HcCht5* were assessed by RT-qPCR at 24 h post injection. (**B**) RNAi silencing of *HcCht5* after co-silencing *HcdsRNase3* and *HcdsRNase4*. The 2-day-old fourth-instar larvae were injected with 6 μg of *GFP-, dsRNase3-*, or *dsRNase4-*dsRNAs or a mixture of 6 μg each of *dsRNase3-* and *dsRNase4-*dsRNAs, after which the insects were injected with 2 μg of dsRNA targeting gene *HcCht5*. Transcription levels of *HcCht5* were assessed by RT-qPCR post 24 h. Values represent the means and standard errors of three biological replicates. Asterisks indicate significant differences according to Student’s *t*-test (* *p* < 0.05; ** *p* < 0.01).

To further assess the in vivo effects of *HcdsRNase* downregulation on RNAi efficacy, the chitinase 5 gene of *H. cunea* (*HcCht5*) was selected as a marker gene, and a double RNAi (RNAi-of-RNAi) experimental setup was used. The relative expression levels of *HcCht5* after the double RNAi treatment of each dsdsRNase plus dsHcCht5 were compared with those when dsGFP plus dsGFP or dsGFP plus dsHcCht5 were injected. The results show that the expression of HcCht5 in dsdsRNase3 plus dsHcCht5 and dsdsRNase4 plus dsHcCht5 group were significantly downregulated compared with the dsGFP plus dsGFP or dsGFP plus dsHcCht5 group (*p* < 0.05) (Figure 6A). However, expression of *HcCht5* in dsdsRNase1 or dsdsRNase2 plus dsHcCht5 group showed no significant difference (Figure 6A). These results demonstrate that knocking down the expression of *HcdsRNase3* and *HcdsRNase4* could significantly enhance the RNAi silencing efficiency of *HcCht5*. We then co-silenced *HcdsRNase3* and *HcdsRNase4* by injecting a mixture of dsdsRNase3 and dsdsRNase4, followed by injection of dsHcCht5. As expected, significantly higher knockdown of *HcCht5* was detected in the co-silencing group than the single HcdsRNase-silenced groups (*p* < 0.05) and the unsilenced control group (*p* < 0.01) (Figure 6B), suggesting that simultaneously knocking down the expression of *HcdsRNase3* and *HcdsRNase4* could enhance the RNAi efficiency of *HcCht5* more effectively. Therefore, we concluded that both *HcdsRNase3* and *HcdsRNase4* can inhibit RNAi efficacy.

## 3. Discussion

Injection and feeding are the basic methods for dsRNA delivery in RNAi experiments, where dsRNA is typically either injected into the body cavity or fed to insects. Thus, the first environment dsRNA encounters prior to cellular uptake is either hemolymph or gut fluid. In many instances, the lack of effective RNAi has been attributed to endogenous nucleases in the hemolymph and gut content that destroy the ingested dsRNAs [31,36]. In general, RNAi is more effective via dsRNA injection than feeding, since dsRNA is particularly unstable in the insect digestive tract [15,16]. However, our previous work showed inefficient RNAi in *H. cunea* regardless of whether dsRNA was delivered by injection or feeding. We speculated that dsRNA degradation by nucleases in the hemolymph and digestive tract might contribute to this low RNAi efficacy. To test this hypothesis, we first extracted hemolymph and gut content for dsRNA stability detection. Unsurprisingly, dsRNA was rapidly degraded in both tissue fluid, even after 10-fold (hemolymph) or 50-fold (gut content) dilution (Figure 1). Similar cases were also observed in other lepidopterans, including *B. mori*, *Manduca sexta*, *S. litura*, *Helicoverpa armigera*, and *H. virescens* [28,31,37,38,39]. Significantly, unlike in most reported insects, dsRNA degradation in hemolymph appears to occur much more rapidly in *H. cunea*, being complete within only 10 min (Figure 1A). dsRNA was reported to be stable in hemolymph in *S. litura* for 1 h, in *B. mori* for 3 h, and in *M. sexta* for 4 h [37,38,39]. Highly rapid degradation of dsRNA in hemolymph might explain why the RNAi efficiency in *H. cunea* is unsatisfactory, and a high dose of dsRNA was needed for injection [20,21]. Further protein expression though specific antibodies might be helpful in verifying the activity of HcdsRNases in hemolymph.

We further identified the *HcdsRNase* genes by examining the *H. cunea* transcriptome database. Four *HcdsRNases* were cloned and characterized by sequencing alignment, domain architecture analysis, and phylogenetic analysis. The results show that HcdsRNases belong to a superfamily of insect nucleases that degrade dsRNA (Figure 2 and Figure 3) [40]. Remarkably, HcdsRNase2 clustered with the Hemiptera insect *Laodelphax striatellus* in the phylogenetic analysis, although HcdsRNase1, 3, 4 had dsRNase homologs in the Lepidoptera clade (Figure S2). Insect dsRNases from the same order did not cluster near each other in all cases [41], suggesting that insect dsRNases are not order-specific. The distribution of HcdsRNases among different insect orders might indicate the functional diversification of *H. cunea* nucleases targeting different nucleic acid substrates.

The expression patterns of *HcdsRNases* in different tissues and life stages of *H. cunea* were detected by RT-qPCR. Results imply that the mRNA levels of *HcdsRNase2, 3,* and *4* increase during the *H. cunea* larval stage, and a significantly high expression level was detected from the third- to fifth-instar larvae (Figure 3A), similar to the development expression pattern in *S. exigua* [42]. dsRNA injection is usually performed on at least third-instar larvae in *H. cunea* to avoid injection-related injuries. The high expression of *HcdsRNases* in older larvae might be one of the reasons for the decreased RNAi efficiency. In addition, the four *HcdsRNase* genes were expressed in almost all the studied tissues in *H. cunea*, predominantly in gut and hemolymph, except that *HcdsRNase1* was not expressed in hemolymph and integument (Figure 3B). Most reported *dsRNases* are specifically expressed either in the intestinal tract or in the hemolymph, such as *PxdsRNase-1, -2, -3* in *Plutella xylostella* [43], *Sg-dsRNases* in *Schistocerca gregaria* [44], and *OndsRNases* in *Ostrinia nubilalis* [41]. In *H. cunea*, extensive expression of *HcdsRNases* in the detected tissues might be the reason for the strong degradation activity of dsRNA in gut content and hemolymph (Figure 1) as well as the low RNAi efficiency in *H. cunea*. Notably, all of the *HcdsRNases* in *H. cunea* showed quite low expression in the head tissues, which might suggest low degradation activity in insect saliva that should be studied in the future. *dsRNases* were mainly expressed in the head and intestine in *Tribolium castaneum* [45], and in the intestine and salivary glands in the *Halyomorpha halys* [46]. In *Acyrthosiphon pisum*, salivary dsRNases were considered to be an important barrier for RNAi efficiency [47]. We speculated that the insect mouthparts and dietary habits might affect the expression of dsRNases in head tissues, which need to be verified in the further study. Overall, our results indicated that the expression of *dsRNases* in different insects is inconsistent.

Notably, dsGFP injection significantly upregulates the expression levels of *HcdsRNase3* and *HcdsRNase4* but not *HcdsRNase1* and *HcdsRNase2* at both 24 and 48 h (Figure 4A,B). RNAi is involved in insect antiviral mechanisms and considered as part of the insect innate immune response [48]. When exogenous dsRNA is injected into the insect body, it is treated as an exogenous pathogen-associated molecular pattern (PAMP) and will be recognized by pattern-recognition receptors (PRRs) to induce an immune response [49]. In *B. mori*, BmdsRNase was regarded as part of a defense mechanism and upregulated after dsRNA injection [37]. In Asian corn borer (*O. furnacalis*), the lepidopteran specific nuclease *REase* was induced upon dsGFP exposure, and suppression of *REase* enhanced RNAi efficiency [28]. In the current study, the upregulation in the expression of *HcdsRNases* by dsGFP injection suggests that *HcdsRNases* might be part of a viral defense mechanism in *H. cunea*, and *HcdsRNase3* and *4* appear to be particularly sensitive to exogenous dsRNA stimulation. The strong immune response of *HcdsRNase3* and *4* might be one of reasons for RNAi inefficiency in *H. cunea*.

In our present study, four *HcdsRNases* were identified in *H. cunea*, though it was confirmed that the inefficiency of RNAi is due to only *HcdsRNase3* and *4* (Figure 6A,B). The case of migratory locust (*Locusta migratoria*) is similar, where although *LmdsRNase2* and *3* were both identified in *L. migratoria* gut, only suppression of *LmdsRNase2* enhanced RNAi efficiency [50]. Wynant, Niels et al. reported four *Sg-dsRNases* in desert locust (*S. gregaria*), but only one was responsible for inefficient RNAi [44]. Katterinne Prentice et al. identified three *dsRNases* in the African sweet potato weevil (*Cylas puncticollis*), and silencing of only *Cp-dsRNase-3* can clearly increase RNAi efficacy [15]. Therefore, not all dsRNases are always involved in the RNAi process in insects, and the involvement of dsRNases in insects is species-specific. dsRNase activity was reported to vary in terms of the optimal reaction conditions and kinetic parameters such as physiological pH or substrate specificity [44,51]. For example, *LmdsRNase1* in *L. migratoria* could efficiently degrade dsRNA at pH 5 and is highly expressed in hemocytes. However, the physiological pH of hemolymph (7.0) severely suppresses *LmdsRNase1* activity, leading to the long-term stability of dsRNA in the hemolymph of *L. migratoria* [16]. Hence, although *HcdsRNases* can be expressed in different tissues, some of them might not necessarily be involved in dsRNA degradation.

As observed in this study, the presence of dsRNases in insect pests is an issue that needs to be addressed because RNAi can be utilized as an effective strategy for pest control. In addition to hemolymph, dsRNA was also rather unstable in the gut content of *H. cunea*, and *HcdsRNases* were highly expressed in the gut tissues (Figure 1C,D and Figure 4B). Whether silencing *HcdsRNases* can improve RNAi efficiency through dsRNA feeding deserves further study, and novel delivery methods mediated by nanoparticles, liposomes, or bacterial expression systems can be explored in future studies to prevent dsRNA degradation and improve RNAi efficiency for control of *H. cunea* and other lepidopteran species [52,53,54]. Besides dsRNases, other nucleases may also be active in dsRNA degradation. The *eri-1* (enhanced RNAi-1) gene, which encodes a nuclease, has been reported to inhibit RNAi efficiency in *Caenorhabditis elegans* [55]. An RNAi efficiency-related nuclease (REase) in Asian corn borer (*O. furnacalis*) was also reported to degrade dsRNA and suppress the RNAi response [28]. Further work on other RNAi efficiency-related nucleases should be researched in *H. cunea*.

In summary, four *dsRNase* genes were identified and characterized in *H. cunea*. Two of the *HcdsRNases*, *HcdsRNase3,* and *HcdsRNase4*, were confirmed to contribute to the inefficient RNAi in *H. cunea*. By knockdown of the nucleases through injection of the insect dsRNase-specific dsRNAs, it was possible to significantly improve RNAi efficacy in this insect. Rapid degradation of dsRNA by HcdsRNases in gut fluids and hemolymph is likely the key factor for the modest RNAi effectiveness observed in our previous study [21]. This work contributes to research on the applicability of RNAi techniques for controlling *H. cunea* and other insect pests.

## 4. Materials and Methods

### 4.1. Insect Rearing

*H. cunea* larvae were kindly provided by the laboratory of Insect Virus Research Center, Chinese Academy of Forestry. They were reared on an artificial diet under a 14 h light/10 h dark photoperiod at 26 (±1) °C with 75% (±10%) relative humidity.

### 4.2. Identification and Characterization of dsRNases from H. cunea

The sequences of *H. cunea dsRNases* were identified in the transcriptome database by the local BLAST program. The amino acid or nucleotide sequences of *dsRNase* genes from *B. mori* (GenBank accessions: XP_028039180.1, XP_004922835.1, and NP_001091744.1) were used as query sequences to search the *H. cunea* transcriptome database using TBLASTN or BLASTN. The retrieved putative cDNA sequences were further confirmed using a BLASTX search against the NCBI nonredundant protein database (https://blast.ncbi.nlm.nih.gov/Blast.cgi, accessed on 19 February 2020). To obtain full-length cDNA sequences of the target genes, rapid amplification of cDNA ends (RACE) was conducted with the SMARTTM RACE cDNA Amplification Kit (Clontech, Palo Alto, CA, USA) using the primers listed in Table 3. The open reading frame (ORF) sequences were further confirmed by PCR amplification using PrimeSTAR^®^ Max DNA Polymerase (Takara, Dalian, China) with the primers listed in Table 3. The PCR products were purified and cloned into a pEASY-Blunt3 vector (TransGen, Beijing, China) and sequenced at the Sangon Biotech Company (Beijing, China).

The molecular weight and isoelectric point (*pI*) of the HcdsRNases were predicted using the Compute pI/Mw tool (https://web.expasy.org/compute_pi/, accessed on 10 March 2020). SMART domain analysis (http://smart.embl-heidelberg.de/, accessed on 10 March 2020) and SignalP 4.1 Server (http://www.cbs.dtu.dk/services/SignalP/, accessed on 10 March 2020) were employed to predict the domain structures and the signal peptides, respectively. The deduced amino acid sequences were aligned by MAFFT (v7.505) [56], and the identities shared among *HcdsRNases* were analyzed by GeneDoc 2.7 (https://genedoc.software.informer.com/, accessed on 25 March 2020).

For phylogenetic relationships analysis, the four HcdsRNase sequences were taken as query sequences, and the BLASTP online tool of NCBI was used to search the whole database. The top sequences with the highest scores were selected as alternative sequences. Then, duplicate sequences were deleted, and only sequences with conserved domains were retained. In addition, eight reported endonuclease G sequences and three reported bacterial nonspecific nucleases were also selected for alignment. A total of 45 amino acid sequences were finally used for tree construction using MEGA 6 software. A maximum likelihood tree was constructed using a JTT substitution model of MEGA 6 under 1000 bootstrap replicates following multiple sequence alignment of deduced amino acid sequences using the Cluster W program in MEGA 6. NCBI accession numbers of protein sequences used to construct the phylogenetic tree are shown in Table S1.

### 4.3. Tissue-Specific and Developmental Expression Analysis

For tissue-specific expression analysis, different tissues (head, gut, fat body, integument, and hemolymph) were dissected from 2-day-old fifth-instar larvae. The larvae were first kept on ice for 3 min and then dissected with a sterile insect scalpel under a zoom stereomicroscope (Olympus, SZX7). The heads from ten larvae and other tissues (gut, fat body, integument, and hemolymph) from three larvae were pooled as one treatment. Each treatment contained three biological replicates. For developmental expression analysis, the whole larva was sampled and used for developmental expression analysis. The first- to fifth-instar larvae (L1 to L5) were collected on the third day of the stadium. Samples from each developmental stage were analyzed as biological triplicates. At least 100 mg of tissues or larvae were sampled per replicate. All collected samples were immediately frozen in liquid nitrogen and stored at −80 °C.

### 4.4. RNA Isolation, cDNA Synthesis, and RT-qPCR

The total RNA was isolated using the TRIzol Plus reagent (Ambion, Austin, TX, USA) following the manufacturer’s recommended protocol. The RNA concentration and quality were assessed using a spectrophotometer (Denovix, Wilmington, DE, USA) and 1% agarose gel electrophoresis. cDNA synthesis was performed by the GoScriptTM Reverse Transcription System kit (Promega, Madison, WI, USA) with an oligo (dT)15 primer, and 1 μg of total RNA was used per reaction. Total RNA and cDNA samples were stored at −80 and −20 °C, respectively.

RT-qPCR was performed using the SuperReal PreMix Plus (SYBR Green) kit (TIAN GEN, Co., Ltd., Beijing, China) with a 20 μL reaction. Each reaction contained the following: 10 μL 2 × SuperReal PreMix Plus (SYBR Green) solution, 0.6 μL forward and reverse primers in a final concentration of 10 μM, 7.8 μL nuclease free water, and 1 μL of undiluted cDNA. RT-qPCR was carried out using a LightCycler 480 II (Roche, Basel, Switzerland) with the following conditions: 95 °C for 3 min, followed by 45 cycles at 95 °C for 5 s, and 60 °C for 30 s. Each treatment included three or four biological replicates and three technical repetitions. *β-actin* is shown to be a good housekeeping gene and is widely applied in RNAi research in *H. cunea* [21]. Therefore, we chose *β-actin* as an internal control for our study. The relative mRNA levels of the target genes were calculated using the 2^−∆∆Ct^ method by normalization of their expression to that of the reference gene. All the PCR primers were designed using Primer Premier 5 software. The primer sequences are listed in Table 3. Melting curve analyses were performed for all the primers.

### 4.5. Synthesis of dsRNA

To obtain dsRNA for the ex vivo incubation experiments and RNAi, the dsRNAs were synthesized using the T7 RiboMAX^TM^ Express RNAi System (Promega, Madison, WI, USA) in accordance with the manufacturer’s instructions. T7 promoter sequences (taatacgactcactatagggaga) were tailed to the 5′-ends of the DNA templates by PCR amplification. GFP was used as a control. The dsRNA primer sequences were designed by targeting the conservative CDS region of each dsRNase gene with a length range between 200 and 400 bp. The primer sequences are listed in Table 3. Template DNA and single-stranded RNA was removed from the transcription reaction by DNase and RNase treatments, respectively. Nuclease-free water (Promega, Madison, WI, USA) was used for dsRNA elution. The dsRNA synthesis was verified by gel electrophoresis and quantified in a spectrophotometer (Denovix, Wilmington, DE, USA).

### 4.6. Incubations of dsRNA in Insect Tissue Extracts

To preparation of insect extracts for ex vivo incubation assays, the last-instar larvae were first starved for 6 h and then dissected on ice with sterile insect scalpels. Using a pipette, about 50–60 μL hemolymph was collected from two individuals after severing their legs. The hemolymph was transferred to an ice-cold tube containing 20 mg phenylthiourea (PTU) to prevent melanization. Samples were then centrifuged at 15,000× *g* for 10 min at 4 °C to remove hemocytes. The supernatant was collected and stored at −20 °C until use. Meanwhile, entire gut tissues from two individuals were transferred to ice-cold tubes containing 100 μL of RNase-free water. Samples were mashed repeatedly with an RNase-free pipette tip to release gut content and then centrifuged at 15,000× *g* for 10 min at 4 °C. The resulting supernatant was collected and stored at −20 °C until use.

To investigate the relative stability of dsRNA in hemolymph and gut content, 6 μL of dsGFP (0.5 μg/μL) was respectively added in the prepared undiluted hemolymph and gut content to final volume of 60 μL and incubated for 10 min, 0.5, 1, 2, 3, and 4 h at 30 °C. To investigate the relative stability of dsRNA in diluted hemolymph and gut content, the prepared hemolymph and gut content were respectively diluted 50- and 10-fold with RNase-free water. An amount of 6 μL of dsGFP (0.5 μg/μL) was respectively added in the diluted extracts to final volume of 60 μL and incubated for 0, 0.5, 1, 2, 3, and 4 h at 30 °C. dsGFP added in RNase-free water was treated as control group. After each time point, the reaction was stored in −20 °C to stop the enzymatic reaction. To visualize dsRNA after incubation, 10 μL of the reaction was loaded onto a 1% (*w*/*v*) agarose gel. dsRNA bands were visualized and photographed using a TGel Image System (TIAN GEN, Co., Ltd., Beijing, China).

For a better evaluation of dsRNA stability in hemolymph, earlier and more intensive time points (2, 5, 10, and 20 min) were set up to examine partial degradation of dsGFP in undiluted hemolymph. 5 μL of dsGFP (2 μg/μL) was added in the undiluted hemolymph to final volume of 60 μL and incubated at 30 °C for the time periods indicated. dsGFP added in RNase-free water was treated as control group. Reactions were then subjected to RNA extraction by TRIzol Plus reagent (Ambion, Austin, TX, USA) following the manufacturer’s instructions. The isolated samples were dissolved in 15 μL RNase-free water and examined (5 μL) on a 1% (*w*/*v*) agarose gel.

### 4.7. Transcriptional Responses of HcdsRNases after dsRNA Injection

To investigate the short-term transcriptional response of *HcdsRNases* after dsRNA injection, four individuals were collected at 24 and 48 h post injection of 6 μg dsGFP into 2-day-old fourth-instar larvae. Whole larvae were used for RNA extraction. Expression analysis of each *HcdsRNase* gene was conducted by RT-qPCR. dsGFP injection was performed using a microinjector (Hamilton, Bonaduz, Switzerland) through the abdominal side between the fourth and fifth abdominal segments of each larva. Larvae injected with the same amount of DEPC water served as controls. To investigate tissue expression levels of *HcdsRNases* after induction by dsGFP, different tissues (gut, hemolymph, and carcass of the larvae) were dissected 24 h after 6 μg dGFP injection of the 3-day-old fourth-instar larvae, and the expression levels of *HcdsRNases* were assayed by RT-qPCR. The injection and dissection methods were the same as described above.

### 4.8. Knockdown of HcdsRNases and RNAi Efficacy Assessment by RNAi-of-RNAi Assays

To evaluate whether silencing of *HcdsRNase* can improve RNAi efficacy in *H. cunea*, the knockdown effect of *HcdsRNase* RNAi and the following improvements of other RNAi targets were tested.

Firstly, 6 μg dsdsRNase was injected into 2-day-old fourth-instar larvae by microinjection, as described above, and RT-qPCR was conducted to detect the expression levels of all the four *HcdsRNase* genes at 48 h post injection. In controls, dsGFP was used instead of dsdsRNase. In each control and treatment, 30 larvae were used, and four individuals (repeats) were collected for RT-qPCR detection after 48 h.

Then, 2 μg dsHcCht5 or dsGFP (control) was injected into the *HcdsRNase*-silenced larvae to measure RNAi efficiency following 48 h post dsdsRNase injection. In total, 20–25 individuals were injected per treatment, and four individuals from each treatment were sampled after 24 h for detection of *HcCht5* expression levels by RT-qPCR.

In addition, injection of a combination of dsdsRNase3 and dsdsRNase4 was conducted to determine their effect on reducing HcdsRNase activity. A total of 20 larvae at 2 days old and of the fourth instar were each injected with 4 μL of dsGFP (6 μg), dsdsRNase3 (6 μg), dsdsRNase4 (6 μg), or a combination of dsdsRNase3 (6 μg) and dsdsRNase4 (6 μg). Then, 2 μg dsHcCht5 was injected after 48 h. The *HcCht5* transcript levels were evaluated by RT-qPCR at 24 h post dsHcCht5 injection. Four individuals (replicates) were sampled from each treatment for RT-qPCR detection. RNA isolation, cDNA synthesis, and RT-qPCR methods were as described above in this section.

### 4.9. Statistical Analysis

For the analysis of *HcdsRNase* expression patterns in different tissues and at different developmental stages, one-way analysis of variance followed by Tukey’s test was applied. The other data were statistically analyzed using an independent sample Student’s *t*-test. In the figures, different letters above the bars represent significant differences in the *HcdsRNase* expression levels between the samples (*p* < 0.05), while asterisks are used to indicate significant differences (* *p* < 0.05; ** *p* < 0.01).

## Figures and Tables

**Table 1:** Properties of NUC proteins from *H. cunea*.

	Amino Acids (a.a.)	Molecular Weight (kD)	Isoelectric Point (*pI*)	Signal Peptide (Position)	NUC Domain (Position)
HcdsRNase1	444	50.56	6.51	Yes (1–18)	Yes (187–400)
HcdsRNase2	329	37.64	9.25	Yes (1–25)	Yes (145–328)
HcdsRNase3	443	49.87	9.00	Yes (1–16)	Yes (186–399)
HcdsRNase4	446	49.99	7.17	Yes (1–16)	Yes (189–402)

**Table 2:** Identities among HcdsRNase protein sequences of *H. cunea*.

	HcdsRNase1	HcdsRNase2	HcdsRNase3	HcdsRNase4
HcdsRNase1	—	34%	70%	68%
HcdsRNase2		—	32%	34%
HcdsRNase3			—	82%
HcdsRNase4				—

**Table 3:** Primer sequences used in this study.

Gene Name		Primer Sequence (5′-3′) ^1^	Product Size (bp)	Primer Usage
*HcdsRNase1*	5′ RACE	CTGCAAGGTGACCTCGGGCCAAGA		RACE-PCR
	3′ RACE	TCCGTGCCCGTATTGGAGCTGCTG		
*HcdsRNase1*	Forward	ATGGGTCTGAGCGTTATG	1335	Full length ORF
	Reverse	TTATGCCAGGAGATCAATAG		
*HcdsRNase2*	Forward	ATGTACACAATGGCCAACGTTTTGA	990	
	Reverse	TTATATGTAAAATAAATATACCTTGTAGAA		
*HcdsRNase3*	Forward	ATGAGTCCGCTTGTTGTATTCGTAG	1332	
	Reverse	TTATGTCAATAGACCAGTGGTGGTG		
*HcdsRNase4*	Forward	ATGGGGCCGCTTGTCGTGTTCGTAG	1341	
	Reverse	TTATGTCAATAGACCAGTGGTGGTGAA		
*HcdsRNase1*	Forward	AAGTTCAGCAGAGGCAAACA	199	RT-qPCR
	Reverse	CAGTTCCAGTTACCAGCATTG		
*HcdsRNase2*	Forward	TAGCTTGCCCTGGAGATAGA	147	
	Reverse	GCTGTGATTTACACCGAATGT		
*HcdsRNase3*	Forward	CAGGACAAATCCGCCTCAAT	142	
	Reverse	CAACCAGCGCCAGACACTAA		
*HcdsRNase4*	Forward	CACCTATCTACGACCCACCG	172	
	Reverse	CCAACCAGAGCCAGACACTAA		
*β-actin*	Forward	GGTTACTCTTTCACCACCACAG	129	
	Reverse	GGACTTCTCAAGGGAACTGC		
*HcCht5*	Forward	TCGGTCGTTCACTTTAGCAG	205	
	Reverse	TTTGTAAGCGTAGGGGCAT		
*dsdsRNase1*	Forward	TTGGTGTTTCGCAACTGCGT	280	dsRNA synthesis
	Reverse	CTGCAGCAGAAGCTGTATCCA		
*dsdsRNase2*	Forward	AACATGCGCAAGCGAAAGTG	328	
	Reverse	TGCACGTGCTGCTAAGTGTCC		
*dsdsRNase3*	Forward	GATAGATATGTCACCAGCCACC	234	
	Reverse	CAGTTGAGTTACCCCGAAGG		
*dsdsRNase4*	Forward	GTGTCTCAACTGCGTGACCA	211	
	Reverse	TGTTGTTGCGACACAGGTCC		
*dsGFP*	Forward	TGAGCAAGGGCGAGGAG	678	
	Reverse	CGGCGGTCACGAACTCCAG		
*dsHcCht5*	Forward	CACGCATCTCATCTACTCA	388	
	Reverse	CGAACCTTTACCGACCCT		

^1^ T7 promoter sequence (taatacgactcactatagggaga) was added in the 5′ end of the primer sequence when dsRNA synthesis.

## Data Availability

GenBank accession numbers are listed for sequence data generated in this study.

## Supplementary Materials

The following supporting information can be downloaded at: https://www.mdpi.com/article/10.3390/ijms23116182/s1.
