# Supplementary material for: RNAi Efficiency through dsRNA Injection Is Enhanced by Knockdown of dsRNA Nucleases in the Fall Webworm, Hyphantria cunea (Lepidoptera: Arctiidae)
Source: Int J Mol Sci. 2022 May 31;23(11):6182. doi: 10.3390/ijms23116182 (PMC9181381; doi:10.3390/ijms23116182)
Supplement: Supplementary file 1 [file ijms-23-06182-s001.zip › sup. figures.pdf]

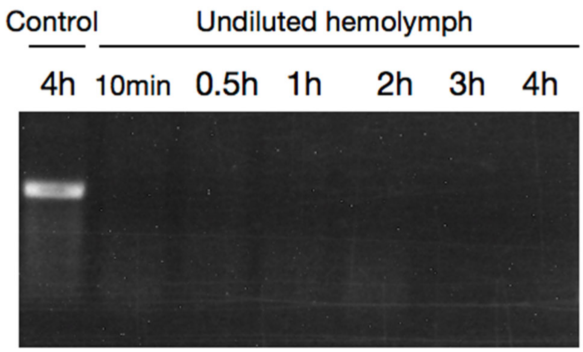

Figure S1. Degradation of dsRNA ex vivo by undiluted hemolymph. 3 μg dsGFP was incubated with undiluted hemolymph at 30° C for 10 min, 0.5, 1, 2, 3, and 4 h, respectively.

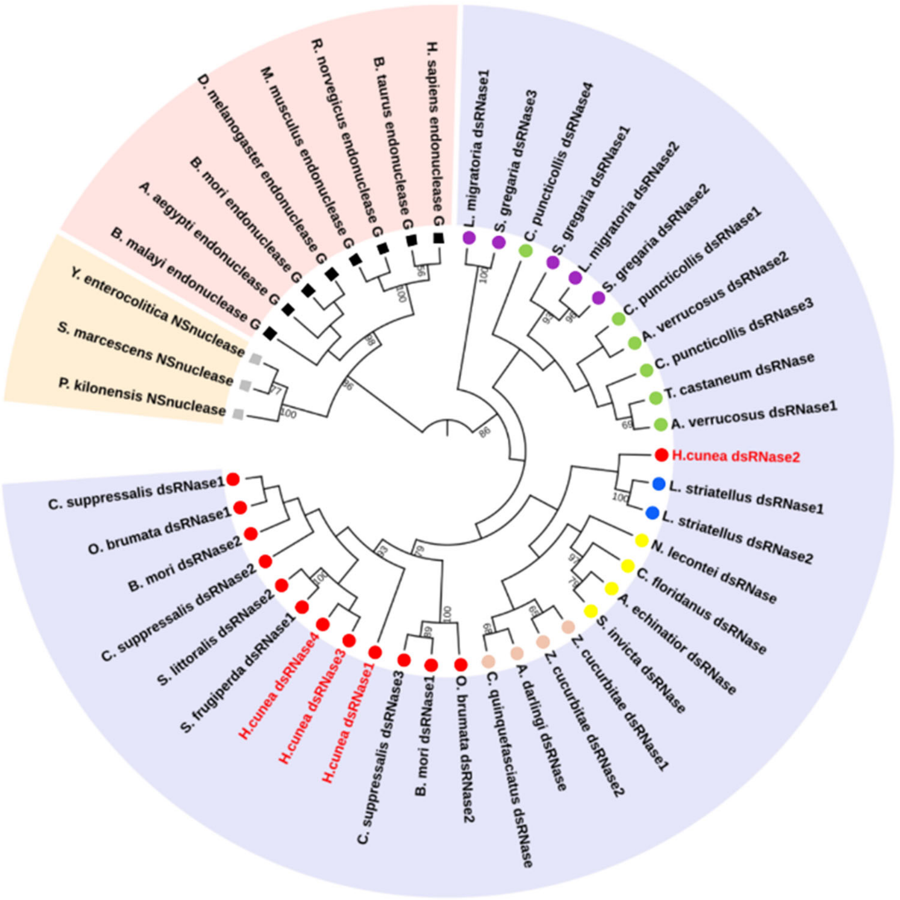

Figure S2. Phylogenetic analysis of HcdsRNases. The phylogenetic tree was constructed by MEGA 6.0 using Maximum Likelihood method. The confidence values (%) of each branch on the phylogenetic trees were calculated based on 1000 bootstrap replicates. Different colors of shadows and shapes indicate the different classifications: purple shadows (insect dsRNase sequences), yellow shadow (bacterial non-specific nuclease sequences, NSnuclease), orange shadow (endonuclease G sequences), red circles (Lepidoptera), pink circles (Diptera), yellow circles (Hymenoptera), blue circles (Hemiptera), green circles (Coleoptera), purple circles (Orthoptera), black squares (eukaryotic endonuclease G), gray squares (NSnuclease). NCBI accession numbers of protein sequences used to construct the phylogenetic tree are shown in Table S1.

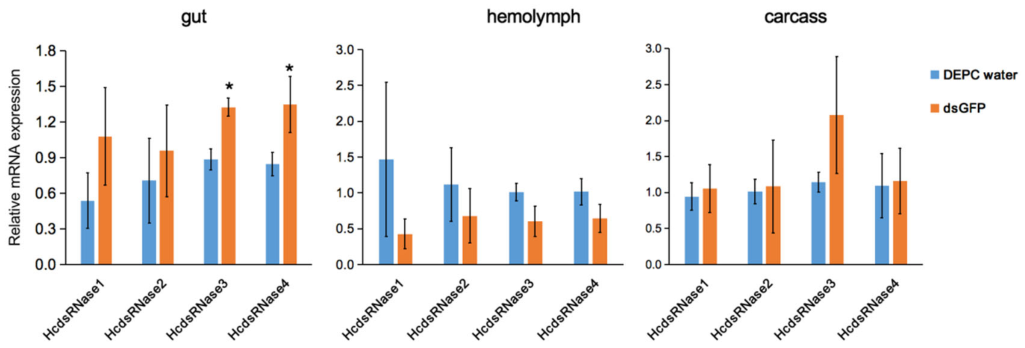

Figure S3. Tissue expression levels of *HcdsRNases* after induction by dsGFP. Third-day-old fourth-instar larvae were injected with 2 µL of 6 µg dsGFP or 2 µL DEPC water (control). Different tissues (gut, hemolymph, and carcass of the larvae) of the treated larvae were dissected 24 h post dsGFP injection. The expression levels of *HcdsRNases* were detected by RT-qPCR. Error bars represent the standard error of the calculated means based on three replicates. Asterisks indicate significant differences ($p < 0.05$).
